# Linking metabolic syndrome with low bone mass through insights from BMI and health behaviors

**DOI:** 10.1038/s41598-023-41513-7

**Published:** 2023-09-01

**Authors:** Chun-Ying Lee, Yun-Shiuan Chuang, Chien-Hung Lee, Ming-Tsang Wu

**Affiliations:** 1 Department of Family Medicine, Kaohsiung Medical University Hospital, Kaohsiung Medical University, Kaohsiung, Taiwan; 2 Research Center for Precision Environmental Medicine, Kaohsiung Medical University, Kaohsiung, Taiwan; 3 Department of Public Health, College of Health Sciences, Kaohsiung Medical University, Kaohsiung, Taiwan; 4 Graduate Institute of Clinical Medicine, Kaohsiung Medical University, Kaohsiung, Taiwan

**Keywords:** Endocrinology, Endocrine system and metabolic diseases

## Abstract

The objective of this study is to investigate the relationship between metabolic syndrome (MetS), and bone density in a 30- to 50-year-old Taiwanese population, and to explore the combined effects of BMI and health behaviors on this association. A total of 52,912 individuals aged 30–50 years from the Taiwan Biobank were included in this cross-sectional study. Bone density status was assessed using quantitative ultrasound (QUS). The joint effect was assessed by including an interaction term in the multi-logistic regression models to test the association between MetS, BMI, and bone density while controlling for potential confounders. MetS was associated with reduced bone density, with the risk of severe low bone density (SLBD) higher among BMI < 24 kg/m^2^ individuals with MetS (adjusted odds ratio [aOR] 1.5, 95% confidence interval [CI] 1.09–2.16), while the risk was not significant among BMI ≥ 24 kg/m^2^ individuals with MetS. Smoking, alcohol consumption, and lack of regular exercise among individuals with a BMI < 24 kg/m^2^ and MetS were associated with higher risk of severe low bone density (SLBD), the aORs (95%CI) were 2.9 (1.59–5.20), 2.1 (1.06–4.22), and 1.8 (1.24–2.54) respectively. Our study suggests that metabolic syndrome could increase the risk of severe low bone density, but this risk can be minimized through higher BMI, non-smoking, no alcohol consumption, and regular exercise. Conversely, smoking, alcohol consumption or lack of regular exercise may exacerbate the risk of severe low bone density. These findings highlight the importance of a multifactorial approach in managing bone healthcare.

## Introduction

Osteoporosis is a skeletal condition characterized by a decrease in the density of mineralized bone, which increases the risk of fracture, disability and mortality^1^. Metabolic syndrome (MetS) is a condition in which cardiometabolic disease risk factors cluster in an individual, increasing the risk of type 2 diabetes, cardiovascular disease and mortality^2^. While osteoporosis and MetS seem unrelated, they share common risk factors, such as aging, cigarette smoking, alcohol consumption, lack of physical activity, and poor dietary habits^3–6^. Both conditions can increase medical costs and impair quality of life^1,2^, indicating that people with these risk factors might have higher risk of co-existing osteoporosis and metabolic syndrome, which can result in a significant socioeconomic burden.

Obesity is a common factor associated with osteoporosis and metabolic syndrome, but in the opposite direction. It is a significant risk factor for metabolic syndrome, in which visceral adiposity plays a central role in the pathogenesis of the development of metabolic syndrome, including insulin resistance, atherosclerotic dyslipidemia, raised blood pressure, and chronic inflammatory state^2^. Conversely, several studies have shown that body mass index (BMI) was positively correlated with bone mineral density (BMD)^7–9^. The traditional explanation is that greater BMI imposes an increased mechanical loading and strain on bone thereby stimulating bone formation; besides, greater amounts of estrogens present in adipose tissue have beneficial effect on preserving bone mass. Studies have also shown that obesity is associated with lower risk of fractures^8,10^, better bone microarchitecture and strength, and lower or unchanged circulating bone resorption, formation and osteocyte markers^8^, which support the positive association between BMI and BMD; nevertheless, body fat mass, the main index of obesity, might not have positive impact on bone mass. Zhao et al. found that fat mass was negatively associated with bone mineral content (BMC) after controlling for the mechanical loading effect by BMI strata^11^. Adipocytokines derived from adipose tissue were found to have metabolic effects on bone, but the effects were not unanimous; for example, adiponectin and resistin were found to be inversely associated with BMD^12–14^, while leptin might have both negative and positive associations with BMD^15^; therefore, the effect of obesity on bone is complex and undetermined.

Evidence of the relationship between metabolic syndrome and osteoporosis or low bone density is inconclusive. Studies have reported that the components of MetS are associated with poor skeletal health, including hyperglycemia^16^, dyslipidemia^17^ and arterial hypertension^18^. Insulin resistance is the key mechanism of developing MetS in obesity; experimental studies have found that insulin could promote the proliferation and differentiation of osteoblasts, which may promote new bone formation^19,20^. Some studies have shown that MetS was associated with increased risk of low bone density^21,22^, while others found MetS was associated with lower risk of osteoporosis^23–25^ or no correlation^26,27^. The complex relationship between the two could involve multiple factors, such as health behaviors. Smoking and excessive alcohol consumption are risk factors for osteoporosis, while regular exercise is a protective factor for osteoporosis. These three factors may also affect body weight, resulting in changes in bone density or metabolism in different aspects. To elucidate the complex association between obesity, MetS and bone density in consideration of health behaviors and to minimize the influence of the ageing process on the study results, we examined the relationship between lifestyle factors, including cigarette smoking, alcohol drinking, exercise, metabolic syndrome and bone density in various degrees of obesity among people aged 30 to 50 from a Taiwan biobank cohort.

## Methods

### Data source and study participants

The Taiwan Biobank (TWB) was established by the Academia Sinica of Taiwan in 2008 as a large-scale, community-based research database that includes adults aged 30 to 70 who were willing to undergo a complete examination. Subjects with prior cancer history were excluded from the database. Further details of the TWB have been described elsewhere^28^. In brief, all participants in the TWB provided informed consent and completed a standardized questionnaire, which elicited information on their dietary habits, personal and familial medical histories, and health behaviors (including smoking status, alcohol consumption and exercise habits) during face-to-face interviews. In addition, the participants underwent a physical examination that included blood pressure and anthropometric and bony density measurements and provided a blood sample. Initially, 122,062 participants from the TWB database recruited between 2008 and 2020 were included. Participants who were older than 50, post-menopausal females, or had self-reported diseases that could cause osteoporosis (e.g. arthritis, thyroid disease, autoimmune disease, renal failure) or incomplete data were excluded, leaving 52,912 participants for further analysis. This study received ethical approval from the Institutional Review Board of Kaohsiung Medical University Hospital (KMUHIRB-E(I)-20190398). All methods were carried out in accordance with relevant guidelines and regulations.

### Definition of overweight and obesity

We used the criteria set by the Ministry of Health and Welfare in Taiwan to define overweight and obesity. These criteria are based on research related to comorbidity, overall mortality rate, and public health epidemiology screening^29^. Being overweight was defined as having a BMI of 24 to < 27 kg/m^2^, while obesity was defined as having a BMI of ≥ 27 kg/m^2^. Normal weight was defined as having a BMI of ≥ 18.5 to 24 kg/m^2^, and underweight was defined as having a BMI of < 18.5 kg/m2. We used these criteria to classify participants in our study.

### Definition of metabolic syndrome

Metabolic syndrome was defined according to the modified National Cholesterol Education Program Adult Treatment Panel III (NCEP ATP III) definition, MetS is diagnosed if at least three of the following five metabolic abnormalities are present: (1) waist circumference ≥ 90 cm for men and ≥ 80 cm for women; (2) triglycerides ≥ 150 mg/dL or pharmacotherapy for hyperlipidemia; (3) HDL cholesterol < 40 mg/dL for men and < 50 mg/dL for women; (4) blood pressure ≥ 130/85 mmHg or pharmacotherapy for hypertension; (5) fasting blood glucose ≥ 100 mg/dL or taking glucose-lowering medication^30^.

### Bone status

While DXA is considered the gold standard for measuring BMD, it has limitations such as radiation exposure, cost and limited availability for field studies. We used quantitative ultrasound (QUS) as a non-invasive, radiation-free alternative to measure bone status in the TWB cohort. The Lunar Achilles Insight ultrasonometer (GE Healthcare, Milwaukee, WI, USA) was used to take measurements at the right calcaneus^31^. QUS analyzes the transmission of sound waves through bone tissue to estimate bone status. The stiffness index (SI) was calculated from the BUA and SOS parameters, and the T-score was estimated based on the SI. The T-score corresponds to the number of standard deviations from the mean value of the population aged 20–39 years of the same sex. Correlations between bone parameters measured by ultrasonometry, BMD, and bone mineral content measured by DXA have been observed in the general population^32^.

In order to investigate the association between bone status and metabolic syndrome, we categorized participants' bone status according to their T-scores. As the World Health Organization defines osteopenia and osteoporosis only in populations over 50 years of age, we used the following classification for our study: T-scores between − 1.5 and − 2.5 were classified as mild low bone density (MLBD), while T-scores ≤ − 2.5 were classified as severe low bone density (SLBD).

### Statistical analysis

Descriptive statistics were used to summarize the demographic characteristics and health information of the study population. Continuous variables were presented as means ± standard deviations (SD), while categorical variables were presented as percentages. Differences in each variable across bone status were tested using either simple regression or logistic analysis. Multi-nominal logistic regression analysis was then performed to investigate the association between metabolic syndrome and bone status. Specifically, Model 1 was formulated to compare the odds values of MLBD and SLBD relative to the normal bone density group. Meanwhile, Model 2 aimed to compare the odds values of SLBD against MLBD. These analyses were adjusting for potential confounding factors including age, gender, educational level, marital status, vegetarianism, cigarette smoking, alcohol drinking, regular exercise and BMI. To examine the joint effect of MetS and overweight/obesity on bone status, we created four categories based on the presence or absence of MetS and overweight/obesity: (1) neither MetS nor overweight/obesity, (2) MetS only, (3) overweight/obesity only, and (4) both MetS and overweight/obesity. The interaction effect between MetS and overweight/obesity was tested by including an interaction term in the multi-nominal logistic regression model. The association further explored the joint effect with cigarette smoking, alcohol consumption and regular exercise, with all statistical analyses being conducted using STATA SE 17.0 (StataCorp LLC, College Station, TX, USA). A p-value < 0.05 was considered statistically significant.

## Results

### Demographic characteristics

A total of 52,912 individuals participated in the study, of whom 38.0% were male, and the mean age was 39.5 ± 5.6 years. The demographic characteristics of the study population according to bone density status are presented in Table 1. The proportion of low bone density was 22.8%, of which 19.5% were MLBD and 3.3% as SLBD. Low bone density was significantly associated with male gender, older age, lower education level, unmarried status, cigarette smoking, alcohol consumption, and lack of regular exercise.

**Table 1:** Demographic characteristics of the study population in relation to bone status.

Factors	Normal	MLBD	Diff.1 ^1^	SLBD	Diff.2^1^	*p*_*trend*_^2^
**Study number**	40,852	10,316		1744		
**Proportion, %**	77.2	19.5		3.3		
**Demographic and risk factors**						
**Age (years), mean ± SD**	39.3 ± 5.6	40.2 ± 5.6	0.9*	40.9 ± 5.5	1.7*	< 0.001
**Male, %**	32.5	54.5		71.1		< 0.001
**Education, %**						
≤ 12 Years	24.3	29.4		34.9		< 0.001
13–16 Years	59.9	56.7		52.6		
≥ 17 Years	15.8	13.9		12.5		
**Married, %**	67.5	69.9	2.4 *	68.7	1.2	< 0.001
**Cigarette smoking, %**	9.8	18.1	8.3 *	26.0	16.2 *	< 0.001
**Alcohol drinking, %**	5.4	8.5	3.1 *	12.0	6.6 *	< 0.001
**Regular exercise, %**	25.4	21.0	− 4.4 *	20.4	− 5.0 *	< 0.001
**Vegetarianism, %**	0.8	0.7	− 0.1	1.2	0.4	0.53
**BMI, mean ± SD**	24.2 ± 4.1	23.9 ± 4.0	− 0.2 *	24.2 ± 4.5	0	< 0.001
**BMI strata**						< 0.001
≤ 18 kg/m^2^, %	2.2	3.5		4.5		
18 ~ 24 kg/m^2^, %	52.1	51.6		49.5		
24 ~ 27 kg/m^2^, %	23.8	24.4		23.7		
≥ 27 kg/m^2^, %	21.9	20.6		22.3		
**MetS component, mean ± SD**						
Waist circumference (cm)	82.2 ± 10.8	83.0 ± 10.9	0.8 *	84.5 ± 11.9	2.3 *	< 0.001
Systolic blood pressure (mmHg)	112.8 ± 15.3	114.9 ± 15.6	2.1 *	117.2 ± 16.0	4.4 *	< 0.001
Diastolic blood pressure (mmHg)	71.3 ± 10.9	73.0 ± 11.5	1.7 *	74.6 ± 11.8	3.3 *	< 0.001
Serum triglyceride (mg/dL)	104.3 ± 94.0	119.3 ± 107.8	15.0 *	132.3 ± 129.1	28.0 *	< 0.001
Serum high-density lipoprotein cholesterol (mg/dL)	54.8 ± 13.2	52.4 ± 13.0	− 2.4 *	50.8 ± 13.2	− 4.0 *	< 0.001
Fasting plasma glucose (mg/dL)	91.5 ± 16.4	92.7 ± 17.5	1.2 *	95.4 ± 26.1	3.9 *	< 0.001
**Self-report disease**						
DM, %	1.6	1.6	0.0	3.0	1.4*	0.006
Hypertension, %	3.5	5.0	1.5*	6.9	3.4*	< 0.001
Dyslipidemia, %	2.4	3.4	1.0*	4.2	1.6*	< 0.001
Cardiovascular disease, %	0.2	0.4	0.2*	0.5	0.3*	< 0.001

Participants with T-score ≥ − 1, − 2.5 ~ − 1, and ≤ − 2.5 were defined as normal, MLBD, and SLBD, respectively.

*MLBD* moderate low bone density, *SLBD* severe low bone density, *BMI* body mass index, *MetS* metabolic syndrome.

*p < 0.05.

^1^Diff.1 and Diff.2 denote the difference in mean or proportion for MLBD vs. normal and SLBD vs. normal, respectively.

^2^*p*_*trend*_ values for increasing or decreasing difference across bone status.

### Association between bone status and metabolic syndrome

**Figure 1:**
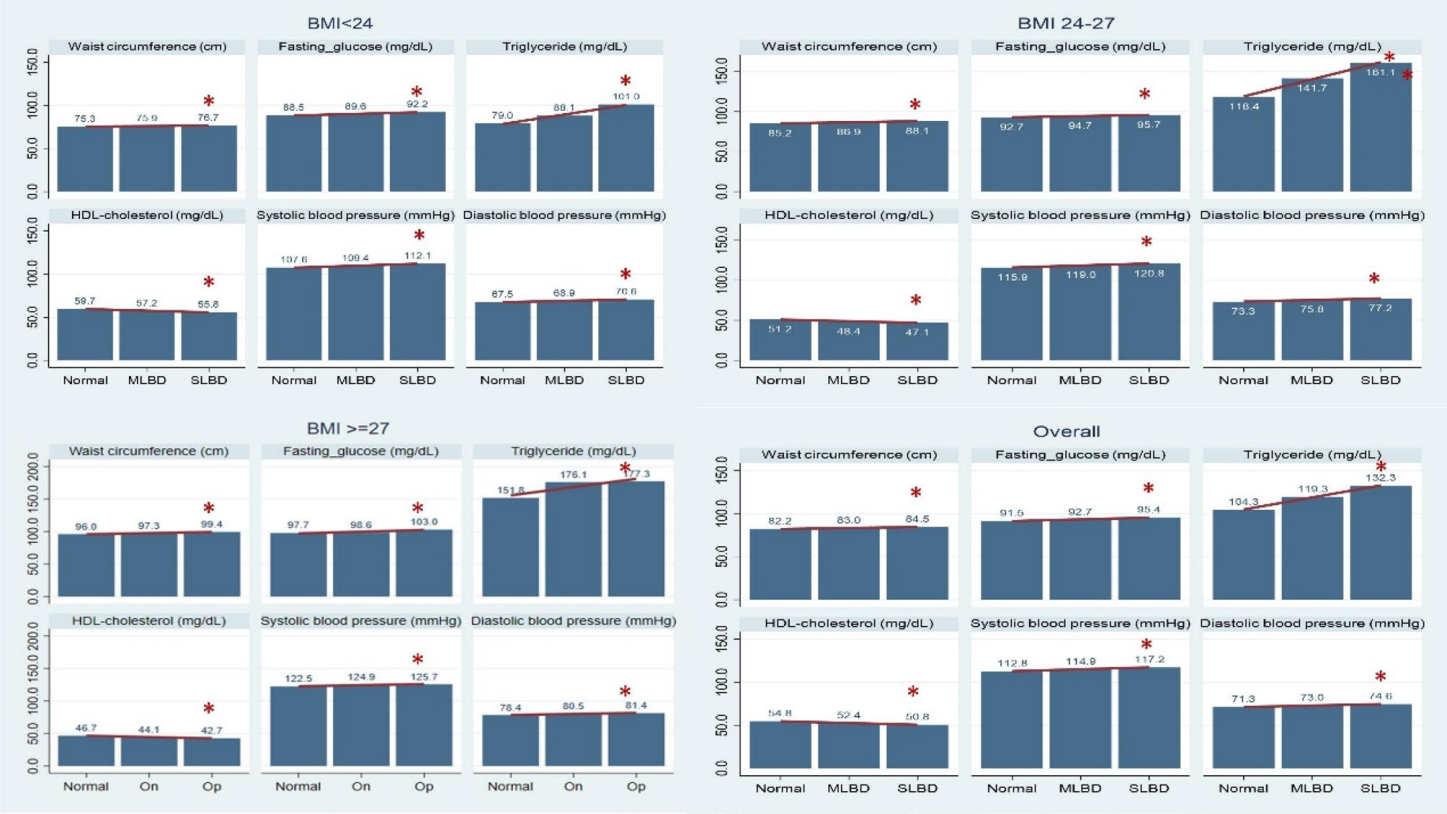
Relationship between components of MetS and bone density status in different BMI strata. The Y-axis represents the value of each metabolic syndrome component, the unit of each factor is indicated in the subtitle of the respective graph. *BMI* body mass index, *MetS* metabolic syndrome, *MLBD* mild low bone density, *SLBD* severe low bone density. *P_*trend*_ < 0.05.

**Figure 2:**
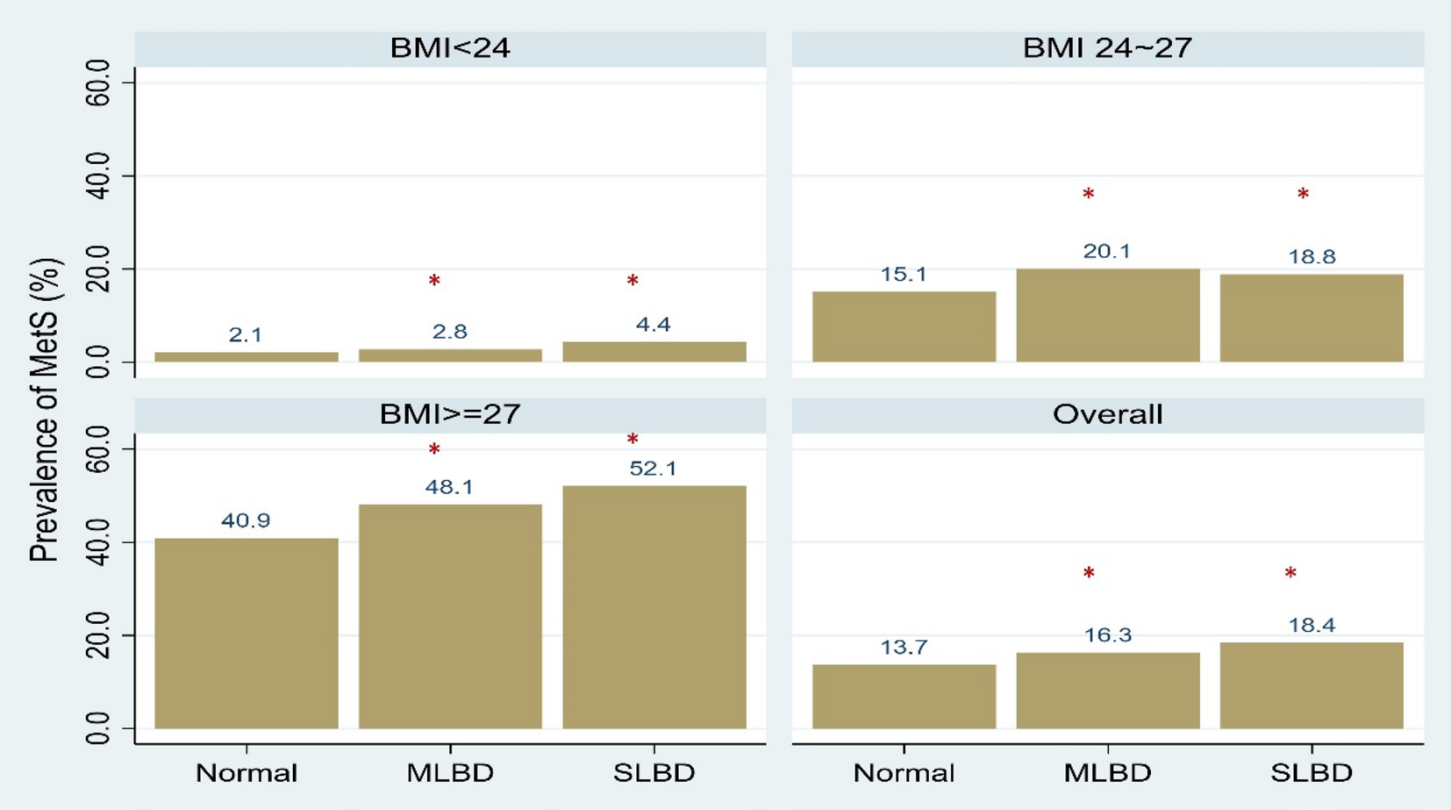
The association of metabolic syndrome and bone density status across different BMI strata. *BMI* body mass index, *MetS* metabolic syndrome, *MLBD* mild low bone density, *SLBD* severe low bone density. *p < 0.05 for comparison to normal bone density group.

Low bone density was associated with unfavorable results of metabolic syndrome components, including larger waist circumference, higher systolic and diastolic blood pressure, higher triglyceride and fasting glucose levels, and lower high-density lipoprotein cholesterol. This association was consistent across different strata of BMI (as shown in Fig. 1). Lower bone density was also associated with presence of self-reported disease, including DM, hypertension, dyslipidemia, cardiovascular disease and having a higher prevalence of MetS, with the results being consistent across different strata of BMI (Fig. 2).

### Effect of BMI on the association between low bone density and metabolic syndrome

To examine the effect of BMI on the association between low bone density and metabolic syndrome, the study population was further categorized based on the presence of overweight/obesity (BMI ≥ 24 kg/m^2^) and MetS with adjusting for potential confounders, including age, gender, educational level, marital status, alcohol drinking, cigarette smoking, regular exercise, vegetarianism and BMI using multi-nominal logistic regression analysis (Table 2). The results showed that among individuals with a BMI < 24 kg/m^2^, those with MetS had a significantly higher risk of SLBD than those without MetS (adjusted odds ratio [aOR] 1.5, 95% confidence interval [CI] 1.09–2.16). On the other hand, among individuals without MetS, those who have a BMI ≥ 24 kg/m^2^ had a significantly lower risk of SLBD compared to those with a BMI < 24 kg/m^2^ (aOR 0.8, 95% CI 0.67–0.92). For individuals with a BMI ≥ 24 kg/m^2^ and MetS, the association with the risk of having SLBD was insignificant (aOR 1.0, 95% CI 0.79–1.21) compared to those with a BMI < 24 kg/m^2^ and without MetS. In the Model 2 analysis, individuals with a BMI ≥ 24 kg/m^2^ also had a significant lower risk of SLBD, irrespective of their MetS status.

**Table 2:** Adjusted odds ratios of bone status associated with BMI strata with or without metabolic syndrome.

BMI and MetS status	Model 1							Model 2	
	Normal	MLBD			SLBD			MLBD vs. SLBD	
	%	%	aOR	(95% CI)	%	aOR	(95% CI)	aOR Ratio	(95% CI)
BMI < 24 with no MetS	53.2	53.5	Ref.		51.7	Ref.		Ref.	
BMI < 24 with MetS	1.1	1.5	1.2	(0.97–1.41)	2.4	1.5*	(1.09–2.16)	1.3	(0.92–1.88)
BMI ≥ 24 with no MetS	33.1	30.2	1.0	(0.92–1.06)	29.9	0.8*	(0.67–0.92)	0.8*	(0.67–0.94)
BMI ≥ 24 with MetS	12.6	14.8	1.3*	(1.15–1.41)	16.0	1.0	(0.79–1.21)	0.8*	(0.62–0.96)

Abbreviations: BMI, body mass index; MetS, metabolic syndrome; MLBD, mild low bone density, denotes T score between − 1.5 and − 2.5; SLBD, severe low bone density, denotes T-scores ≤ − 2.5; Ref., reference; *p < 0.05.

Model 1: Used ‘BMI < 24 with no MetS’ as reference group. The aORs were adjusted for age, gender, educational level, marital status, alcohol drinking, cigarette smoking, regular exercise, being a vegetarian and BMI.

Model 2: Used ‘BMI < 24 with no MetS’ as reference group, and compared the odds of SLBD relative to MLBD. The aORs were adjusted for age, gender, educational level, marital status, alcohol drinking, cigarette smoking, regular exercise, being a vegetarian and BMI.

### Effect of health behaviors on the association between BMI, low bone density and metabolic syndrome

Table 3 presents the effect of cigarette smoking on the association between MetS and SLBD stratified by obesity status. The analysis was adjusting for potential confounders, including age, gender, educational level, marital status, alcohol drinking, regular exercise, vegetarianism and BMI. In general, the risks of SLBD were either insignificant or lower among non-smokers, irrespective of their BMI and MetS status. Among smokers, individuals with a BMI < 24 kg/m^2^, irrespective of MetS status, and those with a BMI ≥ 24 kg/m^2^ in conjunction with MetS, had higher risks of SLBD compared to the reference group.

**Table 3:** Adjusted odds ratios of bone status associated with BMI strata with or without metabolic syndrome and cigarette smoking.

Factors	Model 1							Model 2	
	Normal	MLBD			SLBD			MLBD vs. SLBD	
	%	%	aOR	(95% CI)	%	aOR	(95% CI)	aOR Ratio	(95% CI)
Smoking (−)									
Obesity status with or without MetS									
BMI < 24 with no MetS	49.4	46.3	Ref		40.1	Ref		Ref	
BMI < 24 with MetS	1.0	1.2	1.2	(0.94–1.42)	1.4	1.4	(0.91–2.11)	1.2	(0.77–1.87)
BMI ≥ 24 with no MetS	29.5	24.1	1.0	(0.90–1.05)	22.4	0.8*	(0.68–0.95)	0.8*	(0.69–0.99)
BMI ≥ 24 with MetS	10.3	10.3	1.3*	(1.13–1.40)	10.1	1.0	(0.80–1.28)	0.8	(0.63–1.03)
Smoking (+)									
Obesity status with or without MetS									
BMI < 24 with no MetS	3.8	7.2	1.2*	(1.13–1.38)	11.6	1.6*	(1.33–1.90)	1.3*	(1.06–1.54)
BMI < 24 with MetS	0.1	0.3	1.5	(0.97–2.39)	0.9	2.9*	(1.59–5.20)	1.9*	(1.03–3.48)
BMI ≥ 24 with no MetS	3.6	6.1	1.3*	(1.19–1.51)	7.6	1.2	(0.94–1.90)	0.9	(0.69–1.13)
BMI ≥ 24 with MetS	2.3	4.5	1.6*	(1.42–1.90)	5.9	1.4*	(1.08–1.90)	0.9	(0.65–1.17)

Model 1: Used ‘BMI < 24 with no MetS in non-smokers’ as reference group. The aORs were adjusted for age, gender, educational level, marital status, alcohol drinking, regular exercise, being a vegetarian and BMI.

Model 2: Used ‘BMI < 24 with no MetS in non-smokers’ as reference group. and compared the odds of SLBD relative to MLBD. The aORs were adjusted for age, gender, educational level, marital status, alcohol drinking, regular exercise, being a vegetarian and BMI.

*BMI* body mass index, *MetS* metabolic syndrome, *MLBD* mild low bone density, denotes T score between − 1.5 and − 2.5, *SLBD* severe low bone density, denotes T-scores ≤ − 2.5., *Ref.* reference.

*p < 0.05.

Table 4 presents the effect of alcohol consumption on the association between MetS and SLBD stratified by obesity status. The analysis adjusted for potential confounders, including age, gender, educational level, marital status, cigarette smoking, regular exercise, vegetarianism and BMI. Among non-drinkers, individuals with a BMI ≥ 24 kg/m^2^ and without MetS had lower risk of SLBD compared the reference group. Individuals who were drinkers with a BMI < 24 kg/m^2^ and with MetS had significantly higher risk of SLBD compared the reference group (aOR 2.1, 95% CI 1.06–4.22), while the association was insignificant in non-drinkers.

**Table 4:** Adjusted odds ratios of bone status associated with BMI strata with or without metabolic syndrome and alcohol consumption.

Factors	Model 1							Model 2	
	Normal	MLBD			SLBD			MLBD vs. SLBD	
	%	%	aOR	(95% CI)	%	aOR	(95% CI)	aOR Ratio	(95% CI)
Alcohol consumption (−)									
Obesity status with or without MetS									
BMI < 24 with no MetS	50.9	50.4	Ref		47.0	Ref		Ref	
BMI < 24 with MetS	1.1	1.3	1.1	(0.93–1.39)	1.7	1.4	(0.93–2.04)	1.2	(0.80–1.83)
BMI ≥ 24 with no MetS	31.1	27.3	1.0	(0.91–1.05)	25.8	0.8	(0.65–0.90)	0.8*	(0.66–0.93)
BMI ≥ 24 with MetS	11.5	12.5	1.2*	(1.10–1.37)	13.5	1.0	(0.79–1.22)	0.8	(0.63–1.00)
Alcohol consumption (+)									
Obesity status with or without MetS									
BMI < 24 with no MetS	2.2	3.2	0.9	(0.76–1.00)	4.7	0.9	(0.72–1.19)	1.1	(0.81–1.38)
BMI < 24 with MetS	0.1	0.2	1.2	(0.72–2.14)	0.7	2.1*	(1.06–4.22)	1.7	(0.84–3.43)
BMI ≥ 24 with no MetS	2.0	2.9	1.0	(0.83–1.11)	4.1	0.9	(0.68–1.19)	0.9	(0.69–1.26)
BMI ≥ 24 with MetS	1.1	2.2	1.4*	(1.16–1.67)	2.5	0.9	(0.64–1.33)	0.7	(0.46–0.99)

Model 1: Used ‘BMI < 24 with no MetS in non-alcohol drinkers’ as reference group. The aORs were adjusted for age, gender, educational level, marital status, cigarette smoking, regular exercise, vegetarian status and BMI.

Model 2: Used ‘BMI < 24 with no MetS in non-alcohol drinkers’ as reference group, and compared the odds of SLBD relative to MLBD. The aORs were adjusted for age, gender, educational level, marital status, cigarette smoking, regular exercise, vegetarian status and BMI.

*BMI* body mass index, *MetS* metabolic syndrome, *MLBD* mild low bone density, denotes T score between − 1.5 and − 2.5, *SLBD* severe low bone density, denotes T-scores ≤ − 2.5, *Ref.* reference.

*p < 0.05.

Table 5 shows the effect of regular exercise on the association between MetS and SLBD stratified by obesity status. The analysis adjusted for potential confounders, including age, gender, educational level, marital status, cigarette smoking, alcohol drinking, vegetarianism and BMI. Individuals who had regular exercise and without MetS, regardless of BMI status, had lower risk of SLBD compared to those who did not have regular exercise (aOR 0.7, 95% CI 0.60–0.83 and aOR 0.4, 95% CI 0.33–0.54, for BMI < 24 and ≥ 24 kg/m^2^ respectively). Individuals who had no regular exercise, with BMI < 24 kg/m^2^ and with MetS had higher risk of having SLBD compared to BMI < 24 kg/m^2^ and without MetS (aOR 1.8, 95% CI 1.24–2.54), while the association was insignificant in the regular exercise group (aOR 0.4, 95% CI 0.12–1.23). To better understand the relationship between SLBD and MetS, and the effect of BMI, cigarette smoking, alcohol drinking and exercise on the association, the aORs for SLBD found in Tables 2, 3, 4, 5 is summarized into Table 6.

**Table 5:** Adjusted odds ratios of bone status associated with BMI strata with or without metabolic syndrome and exercise.

Factors	Model 1							Model 2	
	Normal	MLBD			SLBD			MLBD vs. SLBD	
	%	%	aOR	(95% CI)	%	aOR	(95% CI)	aOR Ratio	(95% CI)
Regular exercise (−)									
Obesity status with or without MetS									
BMI < 24 with no MetS	39.4	42.1	Ref		39.9	Ref		Ref	
BMI < 24 with MetS	0.9	1.3	1.2	(0.96–1.46)	2.2	1.8*	(1.24–2.54)	1.5*	(1.03–2.19)
BMI ≥ 24 with no MetS	24.3	23.6	1.0	(0.93–1.09)	24.5	0.8	(0.72–1.00)	0.8	(0.70–1.00)
BMI ≥ 24 with MetS	9.9	12.0	1.2*	(1.12–1.38)	13.1	1.0	(0.78–1.22)	0.8*	(0.62–0.99)
Regular exercise (+)									
Obesity status with or without MetS									
BMI < 24 with no MetS	13.8	11.4	0.7	(0.65–0.76)	11.7	0.7*	(0.60–0.83)	1.0	(0.85–1.19)
BMI < 24 with MetS	0.2	0.3	0.8	(0.48–1.17)	0.2	0.4	(0.12–1.23)	0.5	(0.15–1.70)
BMI ≥ 24 with no MetS	8.8	6.5	0.7*	(0.58–0.72)	5.4	0.4*	(0.33–0.54)	0.6*	(0.50–0.84)
BMI ≥ 24 with MetS	2.7	2.8	1.0	(0.83–1.14)	3.0	0.7	(0.52–1.01)	0.7	(0.52–1.05)

Model 1: Used ‘BMI < 24 with no MetS and no regular exercise’ as reference group. The aORs were adjusted for age, gender, educational level, marital status, cigarette smoking, alcohol drinking, vegetarian status and BMI.

Model 2: Used ‘BMI < 24 with no MetS and no regular exercise’ as reference group, and compared the odds of SLBD relative to MLBD. The aORs were adjusted for age, gender, educational level, marital status, cigarette smoking, alcohol drinking, vegetarian status and BMI.

*BMI* body mass index, *MetS* metabolic syndrome, *MLBD* mild low bone density, denotes T score between − 1.5 and − 2.5, *SLBD* severe low bone density, denotes T-scores ≤ − 2.5, *Ref.* reference.

*p < 0.05.

**Table 6:** Summary of adjusted odds ratios for SLBD associated with metabolic syndrome in condition of BMI status and health behaviors.

		No cigarette smoking	No alcohol consumption	Regular exercise
	aOR	aOR	aOR	aOR
BMI < 24/MetS (−)	–	–	–	No exercise as Ref
BMI < 24/MetS (−)	Ref	Ref	Ref	0.7
BMI < 24/MetS (+)	1.5	NS	NS	NS
BMI ≥ 24/MetS (−)	0.8	0.8	0.8	0.4
BMI ≥ 24/MetS (+)	NS	NS	NS	NS

		Cigarette Smoking	Alcohol consumption	No Regular exercise
	aOR	aOR	aOR	aOR
BMI < 24/MetS (−)	–	Non-smoker as Ref	Non-drinker as Ref	–
BMI < 24/MetS (−)	Ref	1.6	NS	Ref
BMI < 24/MetS (+)	1.5	2.9	2.1	1.5
BMI ≥ 24/MetS (−)	0.8	NS	NS	0.8
BMI ≥ 24/MetS (+)	NS	1.4	NS	0.8

*BMI* body mass index, *MetS* metabolic syndrome, *Ref.* reference, *NS* non-significance.

## Discussion

Our results show that metabolic syndrome was associated with reduced low bone density, with the association being consistent across normal, overweight and obesity groups among people aged 30–50 from a TWB cohort. Subsequent stratification of metabolic syndrome by overweight/obesity (using a BMI cut-off of 24 kg/m^2^) and controlling for the potential confounding factors indicated that normal weight individuals with metabolic syndrome were at an increased risk of lower bone density. Conversely, the risk of lower bone density was attenuated in overweight/obese individuals with metabolic syndrome. Additionally, overweight/obese individuals without metabolic syndrome were found to be at a lower risk of SLBD compared to normal weight individuals.

The results suggest that metabolic syndrome, by itself, could increase the risk of bone loss, and an increased BMI could serve as a protective function in reducing bone loss. Thus, the risk of SLBD in individuals with overweight/obesity and metabolic syndrome is mitigated through the effect of mechanical loading from body weight. A study investigating the association between metabolic syndrome and BMD among adolescents found that metabolic syndrome was associated with a lower concentration of bone biomarkers for osteocalcin, bone alkaline phosphatase and carboxy-terminal telopeptide, indicating reduced bone formation and resorption^33^. Hwang D.K. et al. conducted an analysis of the association between BMD and metabolic syndrome in Korean women aged 18 years and above, with the results revealing that an increased number of metabolic syndrome components was linked to low vertebral BMD^34^. Some studies have previously reported a negative association between MetS and osteoporosis, with this association being diminished after controlling for body mass index (BMI) and other potential confounding factors^26,35^, indicating that the inverse relationship between MetS and osteoporosis might be largely explained by the increased BMI levels observed in individuals with MetS.

The findings of our study provide further evidence supporting this relationship, although in a case–control study that investigated the association between MetS and osteoporosis in adults aged 50 years and above, the findings were inconsistent with our study, demonstrating that MetS was associated with a low occurrence of osteoporosis, but was associated with a high occurrence of osteoporosis among obese men and obese post-menopausal women^24^. One possible explanation for the different findings is that our study population was comprised of younger, pre-menopausal women, suggesting the possibility of a generational difference in the association. Moreover, it is plausible that the duration of obesity and MetS in older populations might have had an impact on bone health; additionally, most studies have examined the effect of MetS and obesity on osteoporosis independently, while our study examined the joint effect of MetS and obesity on severe low bone density, providing an insight into the complex relationship between MetS, obesity and osteoporosis.

Cigarette smoking is recognized as an independent risk factor for the reduction in bone mass and bone length, development of osteoporosis, and increased risk of fractures^36–38^. The effects of cigarette smoking on bone metabolism might involve both indirect and direct mechanisms. Indirect mechanisms could include decreases in body weight, change in the parathyroid hormone-Vitamin D-Axis, gonadal hormones and increased oxidative stress, while other studies have demonstrated that cigarette smoking directly impacts bone tissue by binding to various receptors, such as nicotinic acetylcholine receptors and androgen receptors in osteoblasts, as well as aryl hydrocarbon receptors in osteoclasts^39^.

Our study demonstrated that smokers with normal weight and without MetS, or with MetS regardless of their BMI status, had significantly higher risk of SLBD, when compared to the reference group with normal weight, without metabolic syndrome, and non-smokers. Specifically, individuals with normal weight and metabolic syndrome who smoke had the highest risk of SLBD. These findings suggest that the deleterious effects of smoking on bone health might be more pronounced than the beneficial effects of mechanical loading from body weight; furthermore, smokers are more likely to have lower body weight, which could further exacerbate the negative impact on bone health.

Chronic alcohol consumption, like cigarette smoking, has been found to have both direct and indirect effects on bone metabolism^40^. Animal models have shown that alcohol consumption could disrupt calcium homeostasis through effects on the parathyroid hormone-vitamin D-axis^41^ and growth hormone-insulin-like growth hormone signaling^42^, and inhibit bone formation and fracture repair through several molecular targets^43,44^. The consequences of chronic alcohol consumption on bone include a loss of bone mineral density, impaired bone quality, and an increased risk of osteoporosis^45^ and osteoporotic hip fracture, with a dose–response relationship observed^46^. However, the impact of lower doses of alcohol on bone health remains uncertain. Interestingly, some studies suggest that light drinking might have a beneficial effect on bone mineral density^47^, as it has been observed that BMD is even higher in light drinkers compared to abstainers^46^. Our study demonstrated that alcohol consumption was not significantly associated with SLBD, except in the case of normal-weight individuals with metabolic syndrome, who had significantly higher risk of SLBD. However, the lack of consideration for the amount of alcohol consumed by the participants complicates the interpretation of the results, and accordingly, the association between alcohol consumption and SLBD could not be fully elucidated.

Past studies have reported very consistent results on the beneficial effects of exercise on BMD of the lumbar spine and the femur in both menopausal and more elderly women. Numerous publications have linked physical exercise, bone metabolism markers, and bone mineral density^48^. Our study found that the risk of SLBD was elevated in individuals with normal weight and metabolic syndrome who did not have regular exercise, but was not significant in those who had regular exercise. Additionally, the risk of SLBD was lower in individuals without metabolic syndrome who engaged in regular exercise regardless of their BMI status when compared to the reference group with normal weight, without metabolic syndrome, and non-regular exercisers. Although we did not differentiate the type or intensity of the exercise, these results suggest that, in general, the beneficial effect of regular exercise on bone density could outweigh the negative effect of low BMI and metabolic syndrome.

Our study has several strengths and limitations. Firstly, we utilized a large population-based cohort, enabling us to explore the complex relationship between obesity, metabolic syndrome, health behaviors and bone density. Secondly, we examined the joint effect of metabolic syndrome and obesity, as well as the combined effects of cigarette smoking, alcohol consumption and exercise on bone density, providing additional information on understanding how BMI and health behaviors modify the relationship in early life. However, our study was cross-sectional, which means that a causal relationship between the factors we examined cannot be established, while additionally, our study relied on self-reported data on lifestyle behaviors, which might be subject to reporting bias, with any such bias being non-differentiated, leading to potentially underestimated results. Finally, our study did not consider quantitative information regarding health behaviors such as the amount of smoking and alcohol consumption or the intensity of exercise, which might have important implications for bone health.

## Conclusion

Our study suggests that metabolic syndrome could increase the risk of severe low bone density, but this risk can be minimized through higher BMI, non-smoking, no alcohol consumption, and regular exercise. Conversely, smoking, alcohol consumption or lack of regular exercise could exacerbate the risk of severe low bone density. These findings highlight the importance of a multifactorial approach in managing bone healthcare.

## Data Availability

The data will be provided upon request to the corresponding author.
